# A Novel 10-Point Score System to Predict Early Hematoma Growth in Patients With Spontaneous Intracerebral Hemorrhage

**DOI:** 10.3389/fneur.2019.01417

**Published:** 2020-02-07

**Authors:** Jingjing Fu, Sheng Hu, Mi Yang, Zhaosheng Li, Xiuhua Song, Ziwen Wang, Mar Irida Lloret-Villas, Atlantic D'Souza, Wenbo Xiao

**Affiliations:** ^1^Department of Neurology, School of Medicine, The Fourth Affiliated Hospital of Zhejiang University, Yiwu, China; ^2^Department of Radiology, School of Medicine, The Fourth Affiliated Hospital of Zhejiang University, Yiwu, China; ^3^Division of Neurology, Department of Medicine, University of Alberta, Edmonton, AB, Canada; ^4^Department of Radiology, School of Medicine, The First Affiliated Hospital of Zhejiang University, Hangzhou, China

**Keywords:** spontaneous intracerebral hemorrhage, hematoma growth, CT, score, prediction

## Abstract

**Background:** A rapid and reliable method to predict significant early hematoma growth in the acute setting is of great important to better inform clinicians and researchers in their efforts to improve outcomes for patients.

**Methods:** We established a 10-point score system to predict hematoma growth including four parameters: baseline intracerebral hemorrhage (ICH) volume > 30 mL, time to initial CT scan ≤ 3 h, island sign and black hole sign. Then, we reviewed our ICH database and assessed the predict value of the score system.

**Results:** A total of 216 ICH patients were included. Patients with hematoma growth at 24 h had higher score than those without hematoma growth (7.6 ± 3.0 vs. 2.0 ± 2.4, *p* < 0.001). The optimal cut-off value of the score for predicting hematoma growth was 3 (area under curve, 0.937; 95% CI, 0.899–0.975, *p* < 0.001), with 95% CI of 0.896–0.965 in bootstrapping analysis. The sensitivity, specificity, positive predictive and negative predictive value of the score ≥ 3 for predicting hematoma growth were 97.8, 92.7, 90.9, and 98.3%.

**Conclusion:** The 10-point score system could predict hematoma growth with high accuracy.

## Introduction

Spontaneous intracerebral hemorrhage (ICH) is the most devastating form of stroke and accounts for ~10–30% of strokes worldwide (1). Significant early hematoma growth occurs in about one third of ICH patients who present within few hours and was closely associated with poor outcome and higher mortality (2–4).

A number of prediction score models have been developed to predict early hematoma growth, mainly the 24-point clinical score (BRAIN) (5), 18-point clinical score (6), 9-point clinical score (7), and the value of combining predictors (8). However, these scores demonstrated acceptable discrimination but had some limitations like the requirement of contrast administration and exposure to additional radiation to obtain CTA scan, lack of accuracy, or having a too complex calculation process.

Recently, non-contrast CT (NCCT) imaging signs including island sign, blend sign, and black hole sign showed high accuracy, especially high specificity, in predicting early hematoma growth (9–11). Therefore, based on these NCCT imaging signs, we aimed to establish a simple, accurate, and easy to use score model to predict early hematoma growth.

## Methods

### Patients

This study was approved by the local ethics committee, and all patients had given written informed consent prior to the study. Consecutive adult patients (>18 years) with spontaneous ICH who underwent CT within 6 h after ICH symptom onset and presented in our center between 2015 and 2018 were screened for inclusion into this study. A follow-up CT scan was performed at about 24 h after the initial CT scan. Patients who underwent surgery before the follow-up CT scan or had ICH secondary to arteriovenous malformation, head trauma, cerebral aneurysm, brain tumor, and hemorrhagic transformation of a brain infarction were excluded.

### Imaging Analysis

The admission and follow-up CT scans were performed using standard clinical protocols. Hematoma growth was defined as a 33% increase in hematoma volume or >6 mL at the time of the follow-up CT scan (12, 13). Island sign, blend sign, and black hole sign were defined according to previous literatures (9–11). In detail, the island sign was defined as ≥3 scattered small hematomas all separate from the main hematoma, or ≥4 small hematomas some or all of which may connect with the main hematoma. The blend sign was defined as blending of relatively hypoattenuating area with adjacent hyperattenuating region within the hematoma with a well-defined margin between these regions and a delta of at least 18 Hounsfield units between the 2 regions. The black hole sign was defined as relatively hypoattenuated area (black hole) encapsulated within the hyperattenuating hematoma, and the black hole could be round, oval, or rod-like but was not connected with the adjacent brain tissue, and a delta of at least 28 Hounsfield units between the black hole and other hematoma tissue. Two experienced neuro-radiologists who were blinded to the clinical profiles of the patients reviewed all images independently to assess the presence or absence of the island sign, blend sign, and black hole sign, and discrepancies were settled by consensus.

### Scale Development

Logistic regression was used to investigate associations with ICH growth. Significant predictors (*P* < 0.1) from the univariate analysis were tested for their association with ICH growth in a binary logistic regression model (backward), and odds ratio was used for score development.

### Model Validation

To ensure that the prognostic model is valid, we assessed its discrimination using the area under curve. We used bootstrapping to internally validate the model and repeated this process for 1,000 times.

### Statistical Analysis

Model validation analysis was performed using MedCalc (Version 15.6.1; MedCalc Software bvba). All other statistical analyses were performed using an SPSS software package (Version 22.0; IBM Corporation, Armonk, NY). Fisher's exact test was used to compare categorized variables. Mann–Whitney *U*-test or Kruskal-Wallis *H*-test was used for the continuous variables. The optimal cut-off of admission SBP for predicting hematoma growth was determined by the maximum Youden index. Binary logistic regression analysis was performed using variables that were significantly associated with hematoma growth on univariate analysis. Multicollinearity between variables was checked. Receiver operating characteristic curve analysis was used to determine predictive value. The interobserver agreement for identifying the imaging signs were determined using kappa values. A value of *p* < 0.05 was considered to indicate statistical significance.

## Results

After excluding 31 patients who underwent surgery before follow-up CT scan, a total of 216 patients were included. Table 1 shows the baseline characteristics of included and excluded patients. A total study population of 146 (67.59%) were male, and 70 (32.41%) were female. In total, 74 (34.26%) presented with island sign, 85 (39.35%) with blend sign, and 49 (22.69%) with black hole sign on admission. 92 (42.59%) patients had hematoma growth at 24 h. The interobserver agreement was excellent between the 2 readers for identifying island sign, blend sign, and black hole sign (κ = 0.907, 0.892, 0.910).

**Table 1:** Baseline characteristics of included and excluded patients.

**Variables**	**Included patients (*n* = 216)**	**Excluded patients (*n* = 31)**	***P*-value**
Mean age, years	63.66 ± 14.95	51.55 ± 13.53	<0.001
Male	146 (67.6)	22 (71.0)	0.838
Anticoagulation use	3 (1.4)	0 (0)	1.000
Hypertension	177 (81.9)	28 (90.3)	0.314
Diabetes mellitus	47 (21.8)	5 (16.1)	0.638
Smoking	87 (40.3)	11 (35.5)	0.697
Admission SBP, mmHg	181.55 ± 30.15	180.58 ± 30.05	0.867
Admission DBP, mmHg	102.03 ± 22.33	104.81 ± 22.57	0.519
Time to initial CT scan, hour	2.75 ± 1.38	3.21 ± 1.33	0.082
Hematoma volume, mL	21.90 ± 23.36	25.55 ± 23.90	0.418
Island sign	76 (35.2)	12 (38.7)	0.693
Blend sign	85 (39.4)	14 (45.2)	0.561
Black hole sign	62 (28.7)	10 (32.3)	0.677

SBP, systolic blood pressure; DBP, diastolic blood pressure.

Patients with larger baseline ICH volume, shorter time to initial CT scan, higher admission SBP, island sign, blend sign, and black hole sign are more likely to have hematoma growth at 24 h (all *p* < 0.05, Table 2). The optimal cut-off of admission SBP for predicting hematoma growth is 199 mmHg (area under curve, 0.571; 95% CI, 0.900–0.974, *p* = 0.074). Previous studies use the cut-off of 30 and 60 mL of baseline ICH volume. However, in our data, all patients (*n* = 15) with baseline ICH volume >60 mL had hematoma growth at 24 h. Therefore, we used the cut-off of 30 mL. We use the cut-off of 3 h of time to initial CT scan according to previous studies. The sensitivity, specificity, positive predictive and negative predictive value of island sign for predicting hematoma growth were 72.8, 92.7, 88.2, and 82.1%. The sensitivity, specificity, positive predictive and negative predictive value of blend sign for predicting hematoma growth were 50.0, 68.5, 54.1, and 64.9%. The sensitivity, specificity, positive predictive and negative predictive value of black hole sign for predicting hematoma growth were 57.6, 92.7, 85.5, and 74.7%. Finally, we set anticoagulation use, admission SBP ≥ 199 mmHg, baseline ICH volume > 30 mL, time to initial CT scan ≤ 3 h, island sign, blend sign, and black hole sign in binary logistic regression model (backward), and baseline ICH volume > 30 mL, time to initial CT scan ≤ 3 h, island sign and black hole sign are present in the model (Table 3). Scaling factor was 3.828, and the points are rounded to the nearest integer. No multicollinearity was found.

**Table 2:** Baseline characteristics of 216 patients with spontaneous intracerebral hemorrhage.

**Variables**	**Patients with hematoma growth (*n* = 92)**	**Patients without hematoma growth (*n* =124)**	***P*-value**
Mean age, years	63.72 ± 14.51	63.62 ± 15.33	0.963
Male	60 (65.2)	86 (69.4)	0.558
Anticoagulation use	5 (5.4)	1 (0.8)	0.086
Hypertension	59 (64.1)	76 (61.3)	0.776
Diabetes mellitus	19 (20.7)	28 (22.6)	0.868
Smoking	39 (42.4)	48 (38.7)	0.674
Admission SBP, mmHg	185.48 ± 32.24	176.26 ± 26.34	0.022
Admission SBP ≥ 199 mmHg	16 (17.4)	50 (40.3)	<0.001
Admission DBP, mmHg	103.47 ± 24.50	100.10 ± 18.97	0.274
Time to initial CT scan, hour	2.35 ± 1.19	3.04 ± 1.43	<0.001
Time to initial CT scan ≤ 3 h	82 (89.1)	82 (66.1)	<0.001
Hematoma volume, mL	36.76 ± 25.96	10.87 ± 12.94	<0.001
Hematoma volume >30 mL	49 (53.3)	7 (5.6)	<0.001
Island sign	72 (78.3)	2 (1.6)	<0.001
Blend sign	46 (50.0)	39 (31.5)	0.007
Black hole sign	45 (48.9)	4 (3.2)	<0.001

*SBP, systolic blood pressure; DBP, diastolic blood pressure*.

**Table 3:** Determination of points for each category of each prediction covariate.

**Variables**	**OR**	**95% CI**	***P*-value**	**Points**
Time to initial CT scan ≤ 3 h	6.897	1.818-26.164	0.005	2
Hematoma volume > 30 mL	3.828	1.232-11.895	0.015	1
Island sign	22.844	7.436-70.180	<0.001	6
Black hole sign	5.008	1.676-14.963	0.004	1

The mean score of all patients was 4.2 ± 3.7. Patients with hematoma growth at 24 h had higher score than those without hematoma growth (7.6 ± 3.0 vs. 2.0 ± 2.4, *p* < 0.001). The optimal cut-off value of the score for predicting hematoma growth was 3 (area under curve, 0.937; 95% CI, 0.899–0.975, *p* < 0.001), with 95% CI of 0.896–0.965 in bootstrapping analysis. The sensitivity, specificity, positive predictive and negative predictive value of the score ≥ 3 for predicting hematoma growth were 97.8, 92.7, 90.9, and 98.3%.

## Discussion

We established a novel 10-point clinical score system to predict the risk of early hematoma growth in patients with ICH presenting within 6 h of symptom onset. Our predictive model is based on the following four parameters: baseline ICH volume > 30 mL, time to initial CT scan ≤ 3 h, island sign and black hole sign. Our score system had high sensitivity and specificity when use the cut-off of 3. The sensitivity, specificity, positive predictive and negative predictive value of the score ≥ 3 for predicting hematoma growth were 97.8, 92.7, 90.9, and 98.3%.

The components of the 10-point model are clinical factors and image markers based on NCCT, which were accorded with previous studies. However, in our data, the anticoagulation use was not an independent variable for hematoma growth. The less frequent application of anticoagulation in these patients due to the limited atrial fibrillation ratio might explain this. In previous studies, the sensitivity/specificity value of island sign, blend sign, and black hole sign for predicting hematoma growth were 44.7/98.2%, 39.3/95.5%, and 31.9/94.1%, respectively (9–11). In our data, the sensitivity/specificity value of island sign, blend sign, and black hole sign for predicting hematoma growth were 72.8/92.7%, 50.0/68.5%, and 57.6/92.7%, respectively. Compared with previous studies, the sensitivity value of NCCT signs was higher and the specificity value was lower in our data. The relatively longer time of onset to initial CT scan than previous studies might explain this. The hematoma progressed with time, and more patients present with these signs. In addition, blend sign was not an independent predictor marker for hematoma growth in binary logistic regression model. The simultaneous appearance of blend sign and other two signs might explain this. Further study is needed to explain the different mechanism of these signs.

The strength of our 10-point model was the simplicity of identifying the clinical factors and NCCT markers in all clinical settings. Previous NCCT signs had high specificity value, but their sensitivity value was relatively low. Our model achieved both high sensitivity and specificity after combine two NCCT markers and two clinical factors. Previous 18-point model and 24-point model mainly focused on the clinical variables, rather than image markers, which could explain the relatively low predict values. Besides, 9-point model mainly included CTA spot sign, which limited its clinical application.

There are also several limitations in our analysis. First, our study was a single-institution research that requires validation in other centers. Second, we only included ICH patients within 6 h after the onset of symptoms, and although hematoma growth is known to be less likely to occur after this time, we do not know if our score would also be useful for patients presenting after 6 h. Third, we used odds ratios for score development, rather than beta coefficients from logistic regression, which might come with some problems. A major problem arises when predictors have an odds ratio of 1, reflecting a regression coefficient of zero, that is, no association with the outcome. And the problem would become more apparent when a predictor has a protective or negative effect on the outcome. Although all odds ratio values in our data were higher than 1, the problems should not be ignored. In addition, the odds ratio of each predictor was rounded rather than the corresponding regression coefficient, which also should be cautiously concerned (14). Fourth, due to the limited sample, we did not divide the patients into those used for model production and those used for validation. We used a bootstrapping analysis for validation. Further studies are needed to validate the score model. Finally, the identification of the three novel NCCT markers may be an issue for clinicians not experienced in acute ICH neuroimaging. Further automatic computer aided technology based on these markers may be helpful.

## Data Availability Statement

The datasets analyzed in this article are not publicly available. Requests to access the datasets should be directed to Jingjing Fu, fujingjing1985@zju.edu.cn.

## Ethics Statement

The studies involving human participants were reviewed and approved by Fourth Affiliated Hospital of Zhejiang University, School of Medicine. The patients/participants provided their written informed consent to participate in this study.

## Author Contributions

JF and WX designed the study and collected the clinical data and managed the database. JF, SH, MY, and WX analyzed the data. All authors contributed to draft the manuscript and approved for the manuscript submitted.

### Conflict of Interest

The authors declare that the research was conducted in the absence of any commercial or financial relationships that could be construed as a potential conflict of interest.
